# Interaction between Host MicroRNAs and the Gut Microbiota in Colorectal Cancer

**DOI:** 10.1128/mSystems.00205-17

**Published:** 2018-05-15

**Authors:** Ce Yuan, Michael B. Burns, Subbaya Subramanian, Ran Blekhman

**Affiliations:** a Bioinformatics and Computational Biology Program, University of Minnesota, Rochester, Minnesota, USA; b Department of Surgery, University of Minnesota, Minneapolis, Minnesota, USA; c Department of Biology, Loyola University, Chicago, Illinois, USA; d Masonic Cancer Center, University of Minnesota, Minneapolis, Minnesota, USA; e Department of Genetics, Cell Biology, and Development, University of Minnesota, Minneapolis, Minnesota, USA; f Department of Ecology, Evolution, and Behavior, University of Minnesota, Saint Paul, Minnesota, USA; Oregon State University

**Keywords:** colorectal cancer, gene regulation, microRNA, microbiome, tumor microenvironment

## Abstract

Although variation in gut microbiome composition has been linked with colorectal cancer (CRC), the factors that mediate the interactions between CRC tumors and the microbiome are poorly understood. MicroRNAs (miRNAs) are known to regulate CRC progression and are associated with patient survival outcomes. In addition, recent studies suggested that host miRNAs can also regulate bacterial growth and influence the composition of the gut microbiome. Here, we investigated the association between miRNA expression and microbiome composition in human CRC tumor and normal tissues. We identified 76 miRNAs as differentially expressed (DE) in tissue from CRC tumors and normal tissue, including the known oncogenic miRNAs miR-182, miR-503, and mir-17~92 cluster. These DE miRNAs were correlated with the relative abundances of several bacterial taxa, including *Firmicutes*, *Bacteroidetes*, and *Proteobacteria*. Bacteria correlated with DE miRNAs were enriched with distinct predicted metabolic categories. Additionally, we found that miRNAs that correlated with CRC-associated bacteria are predicted to regulate targets that are relevant for host-microbiome interactions and highlight a possible role for miRNA-driven glycan production in the recruitment of pathogenic microbial taxa. Our work characterized a global relationship between microbial community composition and miRNA expression in human CRC tissues.

**IMPORTANCE** Recent studies have found an association between colorectal cancer (CRC) and the gut microbiota. One potential mechanism by which the microbiota can influence host physiology is through affecting gene expression in host cells. MicroRNAs (miRNAs) are small noncoding RNA molecules that can regulate gene expression and have important roles in cancer development. Here, we investigated the link between the gut microbiota and the expression of miRNA in CRC. We found that dozens of miRNAs are differentially regulated in CRC tumors and adjacent normal colon and that these miRNAs are correlated with the abundance of microbes in the tumor microenvironment. Moreover, we found that microbes that have been previously associated with CRC are correlated with miRNAs that regulate genes related to interactions with microbes. Notably, these miRNAs likely regulate glycan production, which is important for the recruitment of pathogenic microbial taxa to the tumor. This work provides a first systems-level map of the association between microbes and host miRNAs in the context of CRC and provides targets for further experimental validation and potential interventions.

## Abstract (precis)

Recent studies have found an association between colorectal cancer (CRC) and the gut microbiota. One potential mechanism by which the microbiota can influence host physiology is through affecting gene expression in host cells. MicroRNAs (miRNAs) are small noncoding RNA molecules that can regulate gene expression and have important roles in cancer development. Here, we investigated the link between the gut microbiota and the expression of miRNA in CRC. We found that dozens of miRNAs are differentially regulated in CRC tumors and adjacent normal colon and that these miRNAs are correlated with the abundance of microbes in the tumor microenvironment. Moreover, we found that microbes that have been previously associated with CRC are correlated with miRNAs that regulate genes related to interactions with microbes. Notably, these miRNAs likely regulate glycan production, which is important for the recruitment of pathogenic microbial taxa to the tumor. This work provides a first systems-level map of the association between microbes and host miRNAs in the context of CRC and provides targets for further experimental validation and potential interventions.

## INTRODUCTION

The colon microenvironment hosts trillions of microbes, known as the gut microbiome. A healthy microbiome helps maintain colon microenvironment homeostasis, immune system development, gut epithelial function, and other organ functions (1–5). Although many factors impact the composition of the gut microbiome, the overall functional profiles remain stable over time (6, 7). Nevertheless, changes in the taxonomic and functional compositions of the microbiome have been implicated in many diseases, including colorectal cancer (CRC) (8–11). Although the association between microbiome alterations and disease processes has been extensively demonstrated, the directionality, as well as the mediators of the host-microbiome interaction, remains unclear.

Diet has been independently associated with both the gut microbiome and CRC. For example, the Western diet (characterized by low fiber and high protein, fat, and sugar) affects gut microbiome composition in humanized mice, whereby mice fed a Western diet have an increased abundance of *Firmicutes* and a decreased abundance of *Bacteroidetes* (12, 13). The same Western diet has also long been considered a risk factor for developing CRC (14–16). Using an animal model of CRC, Schulz et al. demonstrated that the high-fat diet (HFD) exacerbates CRC progression; however, treating animals with antibiotics blocks HFD-induced CRC progression (17). This suggests that diet can drive microbiome composition change in the gut as a precursor to CRC development.

Recent studies have found that host genetic variation is correlated with microbiome composition. For example, a polymorphism near the *LCT* gene, which encodes the lactase enzyme, is associated with an abundance of *Bifidobacterium* in the gut microbiome, and microbes in the *Christensenellaceae* family were shown to be heritable, with a higher similarity between monozygotic than dizygotic twins (18–23). Another recent study investigated CRC tumors and identified a correlation between coding mutations in tumors and the composition of the microbial community in the tumor microenvironment (24). Interestingly, in a genetic mutation model of intestinal tumors, germfree animals developed significantly fewer tumors in the small intestine (25). Although the finding is limited to the small intestine, the trend shows that CRC development partially depends on the microbiome. In an animal model of colitis-associated CRC, Uronis et al. showed that germfree mice exhibit normal histology and do not develop tumors, compared to 62% of conventionalized mice that developed tumors (*n =* 13) (26). These results support an interaction between the microbiome and host genomics that may affect tumor development.

A recent report demonstrated that fecal microRNAs (miRNAs) can shape the composition of the gut microbiome (27), indicating a mechanism by which host cells can regulate the microbial community. In CRC, several miRNAs, such as miR-182, miR-503, and mir-17~92 cluster, can regulate multiple genes and pathways and have been found to promote malignant transformation and disease progression (28–30). Interestingly, studies have also found that microbiome-derived metabolites can change host gene expression, including expression of miRNAs, in the colon (31, 32). Taken together, these results suggest a bi-directional interaction between host cells and microbes, potentially mediated through miRNA activity. However, we still know very little about the role of miRNAs in host-microbiome interactions, especially in the context of CRC. With thousands of unique miRNAs and microbial taxa present in the CRC microenvironment, it is challenging to experimentally study all possible pairwise interactions. Nevertheless, genomic characterization of both miRNA expression and microbial composition in patients with CRC can identify potential interactions between miRNAs and microbes, which can then be used as candidates for functional inspection.

Here, we establish the relationships between miRNA expression and microbiome composition in CRC patients. We sequenced small RNAs and integrated 16S rRNA gene sequencing data from both tumor and normal colon tissues from 44 patients (88 samples total). We explored the correlation between miRNAs and the microbiome through imputing the miRNA functional pathways and microbiome metabolic pathways *in silico* (see Fig. S1 in the supplemental material). To our knowledge, this is the first analysis to establish a global relationship between miRNA expression and the microbiome in CRC.

10.1128/mSystems.00205-17.1 FIG S1  Overview of analysis. Paired tumor and normal tissues were collected from 44 CRC patients by the University of Minnesota’s BioNet as a part of a previous study. The 16S rRNA gene sequencing was performed earlier, as a part of the previous study. The small RNAs were sequenced on an Illumina HiSeq 2000 platform. A detailed description of the analysis appears in Materials and Methods. Download FIG S1, PDF file, 0.1 MB. Copyright © 2018 Yuan et al. 2018 Yuan et al. This content is distributed under the terms of the Creative Commons Attribution 4.0 International license.

## RESULTS

### MicroRNAs differentially expressed in tumor tissues.

**FIG 1:**
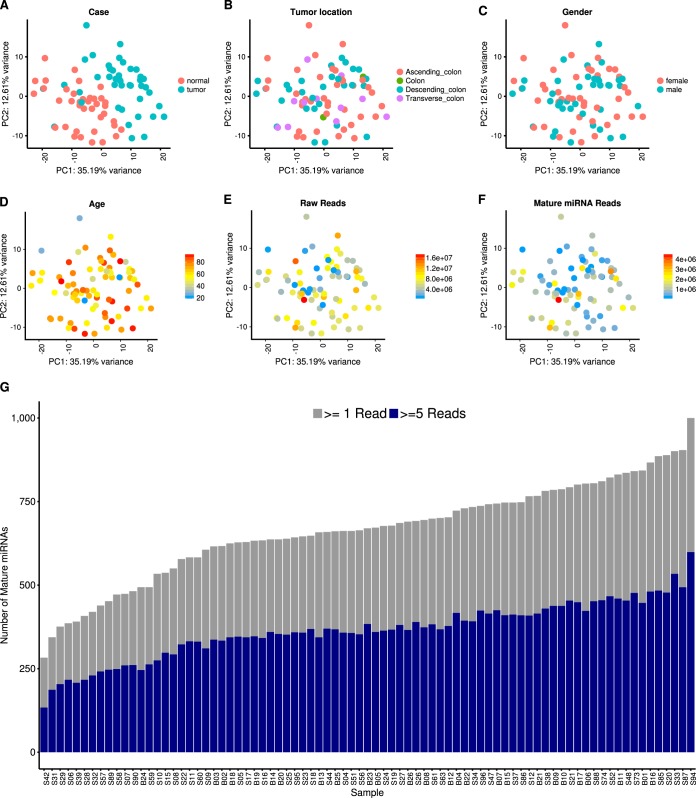
Small RNA sequencing data quality. Principal-component analysis showing principal component 1 (PC1) on the *x* axis and PC2 on the *y* axis. Each dot is colored according to its normal/tumor status (A), tumor location (B), patient gender (C), patient age (D), raw read count (E), and mature miRNA mapped read count (F). (G) Bar plot of the numbers of mature miRNAs identified in each sample, with coverages over 1 read (gray) and over 5 reads (blue).

**FIG 2:**
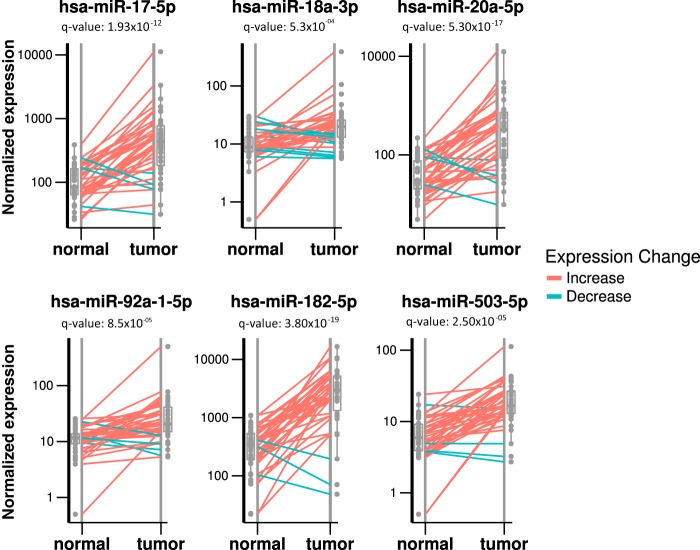
Differentially expressed miRNAs between matched normal and tumor samples. Box plot and dot plot showing differentially expressed miRNAs. Each panel represents a single miRNA with a normalized expression level on the *y* axis. Lines connect a normal and a tumor sample from the same individual, with red lines indicating a higher expression level in tumor tissues and green lines indicating a higher expression level in normal tissues. miR-17, -18a, -20a, -92a, -182, and -503 were found to have significantly higher expression levels in tumor tissues.

Before performing differential expression (DE) analysis, we performed extensive quality control of the miRNA data. Our results indicate that miRNA expression is not strongly affected by tumor location, patient gender, patient age, or read coverage and show a clear clustering of miRNA data by tumor and normal samples (Fig. 1; see also Materials and Methods below). To identify small RNAs that are DE between tumor and normal samples, we performed DE analysis using DESeq2 (see Materials and Methods). A total of 76 DE miRNAs were identified, with 55 upregulated and 21 downregulated in tumor tissues compared to normal tissues (*P* value < 0.05 after false-discovery rate [FDR] correction). A full list of DE miRNAs is available in Table S1 in the supplemental material. DE miRNAs with higher expression levels in tumor tissues include miR-182, miR-183, miR-503, and the miR-17~92 cluster miRNAs (Fig. 2; Table S1), all consistent with our previous reports (28, 33). These miRNAs have all been previously shown to contribute to CRC disease progression; for example, miR-182 and miR-503 were found to cooperatively target *FBXW7* and contribute to CRC malignant transformation and progression and were also predictive of patient survival (28). The miR-17~92 cluster regulates multiple tumor-suppressive genes in CRC and other cancers (34). In addition, miR-1, miR-133a, and miR-448 (Table S1) were observed at higher levels in normal tissues than in matched tumor tissues, also in agreement with previous reports (33, 35).

10.1128/mSystems.00205-17.7 TABLE S1  Full metadata associated with patients and a list of differentially expressed miRNAs. The tab “Full metadata” contains full patient clinical information, including age, gender, tumor location, tumor stage, and microsatellite stability (MSS) status, as well as sequencing quality information. The tab “Differentially expressed miRNAs” contains a full list of differentially expressed miRNAs identified using DESeq2 and for each miRNA includes the identification, log fold change, and *q* value and whether it is more highly expressed in the tumor or normal samples. Download TABLE S1, XLSX file, 0.02 MB. Copyright © 2018 Yuan et al. 2018 Yuan et al. This content is distributed under the terms of the Creative Commons Attribution 4.0 International license.

### Predicted functions of microbiome taxa correlated with DE miRNAs in tumor samples.

**FIG 3:**
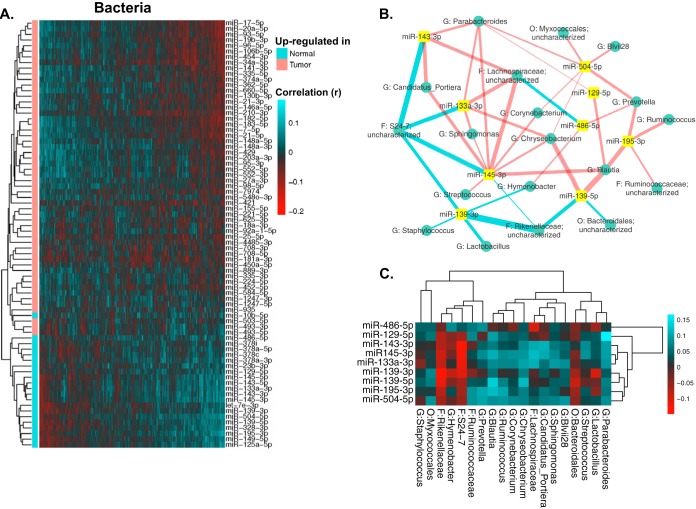
Bacteria significantly correlated with DE miRNAs. (A) Heatmap showing bacterial genera (in columns) that were significantly correlated with the DE miRNAs (in rows). Red indicates negative correlations, and green indicates positive correlations. (B) Interaction network showing the nine most significantly DE miRNAs and their correlated bacteria (showing bacteria with a relative abundance of >0.1% and a correlation pseudo-*P* value of ≤0.05). Edge thickness represents the magnitude of the correlation, with blue indicating negative correlation and with red indicating positive correlation. (C) Heatmap showing the correlations displayed in panel B, with bacterial taxa in columns and miRNAs in rows. Red indicates negative correlations, and green indicates positive correlations.

To investigate the relationship between individual miRNAs and the microbiome in CRC tumor samples, we performed correlation analysis using Sparse Correlations for Compositional Data (SparCC). SparCC is developed specifically to analyze compositional genomic survey data, such as 16S rRNA gene sequencing and other types of high-throughput sequencing data (36). Hierarchical clustering revealed several clusters of significantly correlated miRNAs and bacterial taxa (Fig. S5). To further investigate the relationship between miRNAs and the microbiome in CRC, we selected bacteria significantly correlated with the DE miRNAs (Fig. 3A). The correlations clearly show a distinct pattern based on the enrichment of miRNAs, even though the correlation analysis is performed only with tumor samples. We then built a network visualizing the relationship between the top 9 DE miRNAs and their significantly correlated bacteria (Fig. 3B and C). The correlation network shows a highly interconnected relationship between these miRNAs and bacteria. Interestingly, *Blautia*, a genus previously found to have lower abundance in tumor samples, is negatively correlated with miR-20a, miR-21, miR-96, miR-182, miR-183, and miR-7974, which are all miRNAs with high expression levels in tumor tissues. *Blautia* is also positively correlated with the expression level of miR-139, which is an miRNA with high expression levels in normal tissues. Experimental validations are required to investigate the correlations.

We then analyzed the predicted functional composition of the microbiome data and investigated correlations with miRNAs (Fig. S6). We hypothesized that if miRNAs selectively affect the growth of certain bacteria, then bacteria correlated with DE miRNAs are likely to represent functional differences between tumor and normal tissues, while the uncorrelated bacteria would not. Using the PICRUSt v.1.0.0 software, we generated the predicted functional profiles of the correlated and uncorrelated bacteria by assigning pathways and enzymes using the Kyoto Encyclopedia of Genes and Genomes (KEGG) database. A total of 25 pathways have significantly altered enrichment (two-sided Wilcoxon signed-rank test with an FDR-corrected *P* value of <0.05) (Fig. S6). Interestingly, several metabolic pathways and signaling pathways, including signal transduction, amino acid metabolism, energy metabolism, and linoleic acid metabolism, were all enriched in the uncorrelated group, suggesting increased metabolic processes in this group. For bacteria significantly correlated with DE miRNAs, however, pathways related to transporters, peptidoglycan, and terpenoid backbone biosynthesis have significant enrichment. It is worth noting that the predicted metagenome may not accurately represent the function of the microbiome; further validation using quantitative PCR or high-throughput sequencing is required.

### Predicted functions of miRNAs correlated with CRC-associated bacteria.

**FIG 4:**
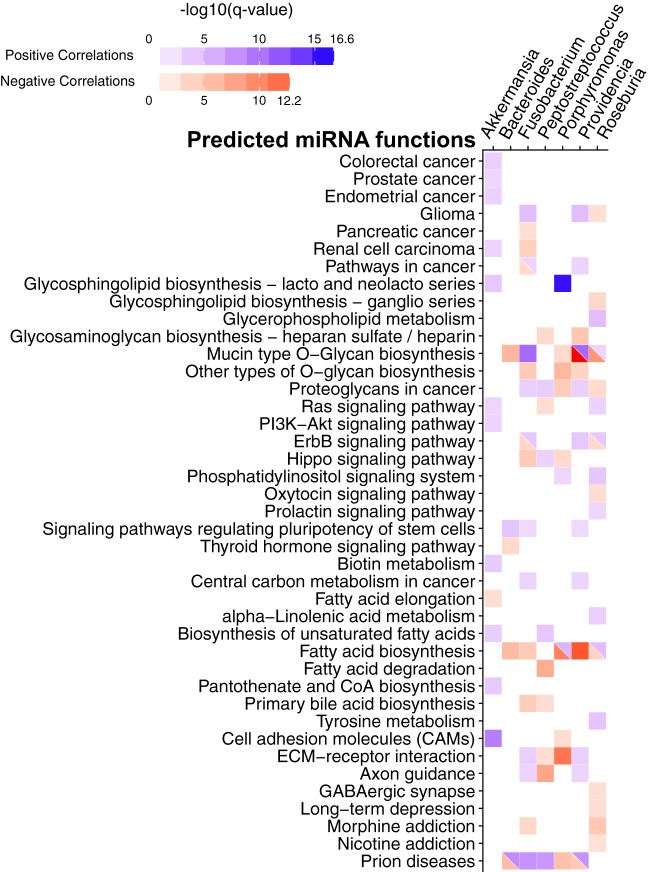
miRNA target pathways correlated with CRC-associated bacteria. The heatmap shows the predicted pathways of miRNAs (rows) correlated with CRC-associated bacteria (columns) with a *q* value of <0.01 (modified Fisher exact test; FDR corrected). Positive correlations are shown in blue, and negative correlations are shown in red. The color intensity is shown in a negative log_10_ scale of FDR-corrected *P* values from a modified Fisher exact test generated by mirPath, with a darker color indicating a lower *q* value. CoA, coenzyme A; ECM, extracellular matrix.

To investigate the function of miRNAs correlated with CRC-associated bacteria, we focused on bacterial genera previously associated with CRC, including *Fusobacterium*, *Providencia*, *Bacteroides*, *Akkermansia*, *Roseburia*, *Porphyromonas*, and *Peptostreptococcus* (8, 37–40). We hypothesized that if these bacteria affect CRC through modulating miRNA expression, then miRNAs that are significantly correlated with the bacteria should show enrichment in cancer-related genes and pathways. A list of miRNAs significantly correlated with these bacteria is available in Table S3. We separated these miRNAs into groups with positive correlation and negative correlation with each bacterium independently. Then, using the miRPath v.3 software, we predicted the functions of miRNAs by assigning pathways to the miRNA targets using the KEGG database (Table S4). We visualized the pathways with a *q* value of <0.01 (modified Fisher exact test; FDR corrected) in Fig. 4.

Our results show that *Akkermansia* is the only taxon correlated with miRNAs associated with the colorectal cancer pathway. *Fusobacterium*, *Providencia*, and *Roseburia* correlate with miRNAs associated with cancer-related pathways, including the glioma, pancreatic cancer, and renal cell carcinoma pathways and pathways in cancer. Interestingly, glycan-related pathways, including the pathways mucin-type O-glycan biosynthesis, other O-glycan biosynthesis, glycosaminoglycan biosynthesis–heparan sulfate/heparin, and proteoglycans in cancer, have correlations with all bacterial genera analyzed, except for *Akkermansia*. This finding corresponds to a previous study showing that Fusobacterium nucleatum infection stimulates mucin secretion *in vitro* (41). Additionally, Fusobacterium nucleatum binds to specific Gal-GalNAc, which is expressed by CRC tumors, through the Fap2 protein (42). Porphyromonas gingivalis was shown to induce shedding of a proteoglycan, syndecan-1, in oral epithelial cells (43). However, the role of the bacteria and glycan interaction is not clear in the context of CRC. Cell signaling pathways previously implicated in CRC, such as the Ras, PI3K/Akt, ErbB, and Hippo pathways, are also correlated with these bacteria (44–47).

## DISCUSSION

Although there is a known association between gut microbiome composition change and CRC (8–11), the potential mediators of this relationship remain unclear. One potential mediator is host genetics and, specifically, CRC tumor mutational profiles (25, 26). Additional evidence indicates that miRNAs can mediate host-microbiome interactions in patients with CRC (27). Here, we presented the first integrated analysis of miRNA expression and gut microbiome profiles in CRC patients. Our data show a highly interconnected correlation network between miRNA expression and the composition of the microbiome and support the role for miRNAs in mediating host-microbiome interactions.

Active interactions between host and the microbiome in CRC have been previously observed, leading to the proposition that pathogenic “passenger” bacteria colonizing tumor tissue might lead to exacerbated tumor progression (48). In our analysis, we focused on potential passenger bacteria, including *Fusobacterium*, *Providencia*, *Bacteroides*, *Akkermansia*, *Roseburia*, *Porphyromonas*, and *Peptostreptococcus*. *Fusobacterium* includes several pathogenic species and is implicated in dental disease, infections, and CRC (49–51). Similarly, *Providencia* has also been implicated in gastrointestinal infections (8, 52–54). The mechanism of *Fusobacterium* in promoting CRC tumorigenesis and progression has been investigated. It activates the *Wnt/β-catenin* signaling pathway through FadA protein, which binds to the E-cadherin protein on intestinal epithelial cells (IECs), thus promoting cell proliferation (49). Several mechanisms might explain this observation. One possibility is that bacteria can infiltrate the intestinal epithelial barrier after certain pathogenic bacteria, cleaving the E-cadherin (49, 55). This might lead to an increased inflammatory response in the colon microenvironment, and the inflammation can lead to DNA damage and contribute to disease progression (48, 49). Another potential mechanism is that bacteria can directly cause mutations in IECs through virulence proteins. Several of these virulence proteins were found in Escherichia coli and Helicobacter pylori (56, 57), and results indicate that these virulence factors may be enriched in the CRC microbiome, especially in *Fusobacterium* and *Providencia* (8). However, it is unclear whether these bacteria produce virulence proteins that can directly cause DNA damage, and further investigation is required to elucidate this mechanism.

The *Wnt/β-catenin* pathway activation by *Fusobacterium* can lead to upregulation of numerous genes related to CRC (58–60). One such gene, *MYC*, is a transcription factor that targets multiple genes related to cell proliferation, the cell cycle, and apoptosis. The miR-17~92 cluster is a known transcriptional target of *MYC* and has oncogenic properties in several cancer types (30, 34, 61, 62). Interestingly, butyrate, a short-chain fatty acid produced by members of the microbiome, diminishes *MYC-*induced miR-17~92 overexpression in CRC *in vitro* through its function as a histone deacetylase inhibitor (31). Studies of CRC have consistently found low fecal butyrate levels as well as a reduced relative abundance of butyrate-producing bacteria, such as members of the *Firmicutes* phylum (31, 37, 63). One potential explanation is that, in CRC, the DE miRNAs can affect the growth of certain microbes, which eventually outcompete other species and form a biofilm on tumor tissues (27). Indeed, our data show several enriched bacterial nutrient biosynthesis and metabolism pathways in the microbes uncorrelated with DE miRNAs, but not in the correlated group. Interestingly, pathways in bacterial cell motility and secretion are also enriched among uncorrelated bacteria, suggesting that, in addition to promoting bacterial growth, certain miRNAs may be involved in recruiting bacteria to tumor tissues. This may also provide a possible explanation for the observed difference in alpha diversity of tumor microbiomes (8, 64, 65).

In our analysis of the functions of miRNAs correlated with selected bacteria known to have associations with CRC, prion disease, and morphine addiction pathways found to be enriched in our analysis do not immediately seem related to cancer (Fig. 4). Upon further investigation of miRNA target genes in these pathways, we found that several genes included in the pathways may have relevant functions in cancer. For example, mitogen-activated protein kinase (*MAPK*) is central to cell proliferation and survival, interleukin-6 (*IL6*) and interleukin-beta (*IL1β*) are cytokines involved in inflammation, protein kinase A (*PKA*) is important in regulating nutrient metabolism, Bcl-2-associated X protein (*BAX*) is a tumor suppressor gene; and prion protein (*PRNP*) are known to have a significant role in regulating immune cell function (66–68).

A recent study has suggested an additional mechanism affecting host-microbiome interactions that may promote CRC tumorigenesis and progression (42, 69). Abed et al. showed that Fap2 produced by *Fusobacterium* binds to glycan produced by CRC to attach to the tumor tissue (42). Interestingly, glycan biosynthesis pathways were enriched in targets of the miRNAs correlated with CRC-associated bacteria. The increased glycan production may increase recruitment of certain bacteria, such as *Fusobacterium*, to the tumor location. This result highlights a novel potential mechanism for miRNAs, through regulating glycan biosynthesis, to attract specific microbes to the tumor microenvironment and thus impact tumor development. Interestingly, the mucin-type O-glycan biosynthesis pathway is enriched in miRNAs positively correlated with *Fusobacterium* but negatively correlated with *Bacteroides* and *Porphyromonas*. This suggests that these bacteria may have different mechanisms of attachment to the mucosal surface due to different abilities to bind to O-glycan (70). Additional studies are required to test the association between *Fusobacterium*, tumorigenesis, and miRNA-driven glycan production.

It is important to note that our study uses 16S rRNA gene sequencing to characterize microbiome taxonomic composition and computationally predicted pathway composition using PICRUSt v1.0.0 (71). Although this method is widely used, metagenomic shotgun sequencing can be more accurate and informative in understanding the functional makeup of a microbial community. Similarly, to impute miRNA functional profiles, we used an *in silico* prediction method, miRPath (71, 72). While both of these methods have been rigorously tested and validated with experimental data, the results remain predictions and may not represent the real biological system (71, 72). Another limitation of our approach is that it identifies correlations and not causal relationships. Nevertheless, this approach allows us to generate a microbiome- and miRNA transcriptome-wide characterization of potential interactions, which shed light on potential new mechanisms of host-microbiome interactions.

In addition, we highlight candidates for potentially interacting host miRNAs and microbial taxa, which can be directly validated and explored in model systems (73). For example, mouse models have been extensively used to study host-microbiome interactions in the gut (74), and studies have quantified how microbiome colonization can modulate gene expression in the host gut (75, 76). In addition, *in vitro* approaches can be useful in dissecting the regulatory effects of the microbiome and in characterizing the effects of variation in individual taxon abundances on gene expression in host cells (77, 78). These studies can validate interactions identified in our current study and shed light on the directionality and causality.

### Conclusions.

Our analysis, together with evidence from previous studies, suggests that miRNAs likely mediate host-microbiome interaction in CRC. We identify potential novel mechanisms that mediate this interaction and may have a role in CRC tumorigenesis, including a possible role for miRNA-driven glycan production in the recruitment of pathogenic microbial taxa. The interactions identified here might be a direct target for developing therapeutic strategies that can benefit CRC patients. Follow-up studies using model systems are warranted to assess the causal role of individual microbes and miRNAs in CRC.

## MATERIALS AND METHODS

### Tissue samples.

A total of 88 matched tumor and adjacent normal tissues were collected from 44 patients by the University of Minnesota Biological Materials Procurement Network. A detailed description of sample collection was previously published (8). Briefly, all patients provided written, informed consent. All research conformed to the Helsinki Declaration and was approved by the University of Minnesota Institutional Review Board, protocol 1310E44403. Tissue pairs were resected concurrently, rinsed with sterile water, flash frozen in liquid nitrogen, and characterized by staff pathologists. Detailed deidentified sample metadata, including age, gender, tumor location, tumor stage, and microsatellite stability (MSS) status, are available in Table S1 in the supplemental material.

### 16S rRNA sequencing and sequence analysis.

The 16S rRNA gene sequencing data were previously published (8). Raw sequences were deposited in the NCBI Sequence Read Archive under project accession number PRJNA284355, and processed data files are available in the work of Burns et al. (8). Briefly, total DNA was extracted from approximately 100 mg of tissue. Tissues were first physically disrupted by placing the tissue in 1 ml of QIAzol lysis solution in a 65°C ultrasonic water bath for 1 to 2 h. The efficiency of this approach was verified by observing high abundances of Gram-positive bacteria across all samples, including those from the phylum *Firmicutes*. DNA was then purified using an AllPrep nucleic acid extraction kit (Qiagen, Valencia, CA). The V5-V6 region of the 16S rRNA gene was PCR amplified with multiplexing barcodes (79). The bar-coded amplicons were pooled and ligated to Illumina adaptors. Sequencing was performed on a single lane on an Illumina MiSeq instrument (paired-end reads). The forward and reverse read pairs were merged using the USEARCH v7 program fastq_mergepairs, allowing stagger but no mismatches (80). Operational taxonomic units (OTUs) were picked using the closedreference picking script in QIIME v1.7.0 and the Greengenes database (August 2013 release) (81–83). The similarity threshold was set at 97%, reverse read matching was enabled, and reference-based chimera calling was disabled. The unfiltered OTU table used for the analysis is available in Table S2.

10.1128/mSystems.00205-17.8 TABLE S2  Unfiltered OTU table with relative abundances of the taxa used in the analysis. Download TABLE S2, TXT file, 0.5 MB. Copyright © 2018 Yuan et al. 2018 Yuan et al. This content is distributed under the terms of the Creative Commons Attribution 4.0 International license.

10.1128/mSystems.00205-17.9 TABLE S3  List of miRNAs significantly correlated with bacteria previously associated with CRC. Each sheet includes the miRNAs correlated with one of the seven taxa (*Fusobacterium*, *Providencia*, *Bacteroides*, *Akkermansia*, *Roseburia*, *Porphyromonas*, and *Peptostreptococcus*) and provides the miRNA’s identity, correlation coefficient, and pseudo-*P* value. Download TABLE S3, XLSX file, 0.04 MB. Copyright © 2018 Yuan et al. 2018 Yuan et al. This content is distributed under the terms of the Creative Commons Attribution 4.0 International license.

10.1128/mSystems.00205-17.10 TABLE S4  Predicted functions of miRNAs correlated with CRC-associated bacteria. Each sheet represents the predicted functions of miRNAs correlated with one of the taxa listed in the sheet’s name. Download TABLE S4, XLSX file, 0.02 MB. Copyright © 2018 Yuan et al. 2018 Yuan et al. This content is distributed under the terms of the Creative Commons Attribution 4.0 International license.

### MicroRNA sequencing.

To prepare samples for small-RNA sequencing, total RNA was extracted using an AllPrep nucleic acid extraction kit (Qiagen, Valencia, CA). RNA was quantified using the RiboGreen fluorometric assay (Thermo Fisher, Waltham, WA). RNA integrity was then measured using a model 2100 Bioanalyzer (Agilent, Santa Clara, CA). Library creation and sequencing were performed by the Mayo Clinic Genome Analysis Core. Briefly, small-RNA libraries were prepared using 1 µg of total RNA per the manufacturer’s instructions for the NEBNext multiplex small-RNA kit (New England Biolabs, Ipswich, MA). After purification of the amplified cDNA constructs, the concentration and size distribution of the PCR products were determined using an Agilent (Santa Clara, CA) Bioanalyzer DNA 1000 chip and Qubit fluorometry (Invitrogen, Carlsbad, CA). Four of the cDNA constructs are pooled, and the 120- to 160-bp miRNA library fraction is selected using Pippin Prep (Sage Science, Beverly, MA). The concentration and size distribution of the completed libraries were determined using an Agilent Bioanalyzer DNA 1000 chip and Qubit fluorometry. Sequencing was performed across 4 lanes on an Illumina HiSeq 2000 instrument (paired end).

### MicroRNA sequence data processing and QC.

See Fig. S1 for an overview of the data analysis steps. Briefly, quality control (QC) of miRNA sequencing data was performed using FastQC before and after adaptor trimming with Trimmomatic (84). Then, the paired-end reads were assembled using PANDAseq and aligned to the hg38 genome assembly using bowtie2 (85, 86). Finally, the total mature miRNA counts were generated with HTSeq (87). We removed 7 samples (S01, S02, S03, S36, S40, S41, S43) due to a low number of total raw reads (fewer than 500,000 raw reads) from the analysis (Table S1). A previous study showed that a number of miRNA sequencing reads as low as 500,000 provides sufficient coverage for analysis (88). The remaining 81 samples have between 519,373 and 17,048,093 (median, 6,010,361) reads per library, with an average quality score of greater than 37 in all libraries. Between 66.79% and 96.14% (median, 83.53%) of reads passed adapter trimming (Fig. S2). Of all the reads passing adapter trimming, between 287,356 and 11,102,869 (median, 3,701,487) reads were identified as concordant pairs by PANDAseq. After being mapped to the hg38 genome, between 18,947 and 4,499,805 (median, 859,546) reads were assigned to a total of 2,588 mature miRNAs (Fig. S2). Principal-component analysis (PCA) visually shows a clear separation between tumor and normal samples (Fig. 1A), while tumor location, gender, age, total raw reads, and total mature miRNA reads do not appear to have an impact on the data (Fig. 1B to F). Similarly, PCA plots, including an additional principal component, did not detect clustering based on these factors (Fig. S3). We further performed discriminant analysis of principal components (DAPC) using the adegenet package in R, and it confirmed the existence of separate clusters for tumor and normal samples (*P* < 2.2 × 10^−16^) (see Fig. S4) but not for gender and tumor locations (*P* > 0.2 for all comparisons) (Fig. S4) (89). Between 283 and 1,000 (median, 670) miRNAs had coverage over 1 read, and between 134 and 599 (median, 367) miRNAs had coverage over 5 reads (Fig. 1G). Overall, the quality of our sequencing results is on par with those of previous studies and our previous observations (90).

10.1128/mSystems.00205-17.2 FIG S2  Bar plots showing quality control of raw reads. (A) Total numbers of raw reads on a log_10_ scale per sample (gray) and percentages of reads surviving quality control (blue); (B) percentages of reads surviving quality control per sample; (C) bar plot of percentages of concordant paired-end reads per sample; (D) total numbers of mapped mature miRNAs per sample on a log_10_ scale. Download FIG S2, PDF file, 0.2 MB. Copyright © 2018 Yuan et al. 2018 Yuan et al. This content is distributed under the terms of the Creative Commons Attribution 4.0 International license.

10.1128/mSystems.00205-17.3 FIG S3  Principal-component analysis (PCA) plot showing PC1 on the *x* axis and PC3 on the *y* axis. Each dot was colored according to case (A), tumor location (B), gender (C), age (D), raw reads (E), and mature miRNA reads (F). Download FIG S3, PDF file, 0.1 MB. Copyright © 2018 Yuan et al. 2018 Yuan et al. This content is distributed under the terms of the Creative Commons Attribution 4.0 International license.

10.1128/mSystems.00205-17.4 FIG S4  Discriminant analysis of principal components (DAPC) using the first 3 principal components. (A) Case status shows a clear separation between the tumor and normal groups. Genders (B) and tumor location (C) do not appear to significantly impact miRNA expression. Statistical significances are tested using the two-sample Kolmogorov-Smirnov test, and multiple comparisons are adjusted using the false-discovery rate (FDR) method. Download FIG S4, PDF file, 0.1 MB. Copyright © 2018 Yuan et al. 2018 Yuan et al. This content is distributed under the terms of the Creative Commons Attribution 4.0 International license.

10.1128/mSystems.00205-17.5 FIG S5  Heatmap of miRNA-microbiome correlations. The correlations were estimated using SparCC. Bacterial taxa are shown in the column, and miRNAs are in the rows. Red indicates negative correlations, and green indicate positive correlations. Download FIG S5, JPG file, 0.2 MB. Copyright © 2018 Yuan et al. 2018 Yuan et al. This content is distributed under the terms of the Creative Commons Attribution 4.0 International license.

10.1128/mSystems.00205-17.6 FIG S6  Metabolic pathway (KEGG) enrichment of microbiomes correlated and uncorrelated with DE miRNAs. The bar graph (left panel) shows the fold enrichment for each group. Red indicates correlated and blue indicates uncorrelated KEGG enrichment. FDR-corrected *P* values from a Wilcoxon rank-sum test (on a negative log_10_ scale) are shown in the right panel. The solid red line indicates a *q* value of 0.05. Download FIG S6, PDF file, 0.1 MB. Copyright © 2018 Yuan et al. 2018 Yuan et al. This content is distributed under the terms of the Creative Commons Attribution 4.0 International license.

### MicroRNA differential expression and correlation analysis.

We identified differentially expressed (DE) miRNAs between tumor and normal samples using the DESeq2 package (1.10.1) in R (version 3.2.3) (91). Raw miRNA counts were filtered to include miRNAs with ≥1 read in ≥80% of the samples. The remaining 392 miRNAs were then used for DESeq2 analysis. We define DE miRNAs as showing a fold change of over 1.5, with a false-discovery rate (FDR)-adjusted *P* value (*q* value) of <0.05. We performed correlation analysis for the tumor samples using Sparse Correlations for Compositional Data (SparCC) at the genus level for bacteria and the miRNAs (36). To increase the accuracy of estimation, we performed 20 iterations for each SparCC procedure. SparCC then performs 100 permutations to calculate the pseudo-*P* values. Significant correlations were defined as a correlation coefficient (*r*) of over 0.05 (or less than −0.05), with a pseudo-*P* value of ≤0.05 (8). Heatmaps of the correlation were generated in R using the pheatmap package. We performed hierarchical clustering for both columns and rows with the average linkage method using Pearson’s correlation. We utilized PICRUSt v1.0.0 to construct a predicted metagenome for bacteria with significant correlations with the DE miRNAs in tumor tissues (71). Specifically, bacterial OTUs that are significantly correlated with the DE miRNAs are collapsed to the species level (L7). The predicted metagenomes are then generated by following the standard PICRUSt metagenome prediction pipeline. We included miRNAs with significant correlations with CRC-associated genera (*Fusobacterium*, *Providencia*, *Bacteroides*, *Akkermansia*, *Roseburia*, *Porphyromonas*, and *Peptostreptococcus*) to perform pathway enrichment analysis using miRPath v.3 (72, 92). We generated network visualization of miRNA-microbe using Cytoscape v3.5.1.
