# Supplementary material for: Interaction between Host MicroRNAs and the Gut Microbiota in Colorectal Cancer
Source: mSystems. 2018 May 15;3(3):e00205-17. doi: 10.1128/mSystems.00205-17 (PMC5954203; doi:10.1128/mSystems.00205-17)
Supplement: FIG S1 [file sys003182230sf1.pdf]

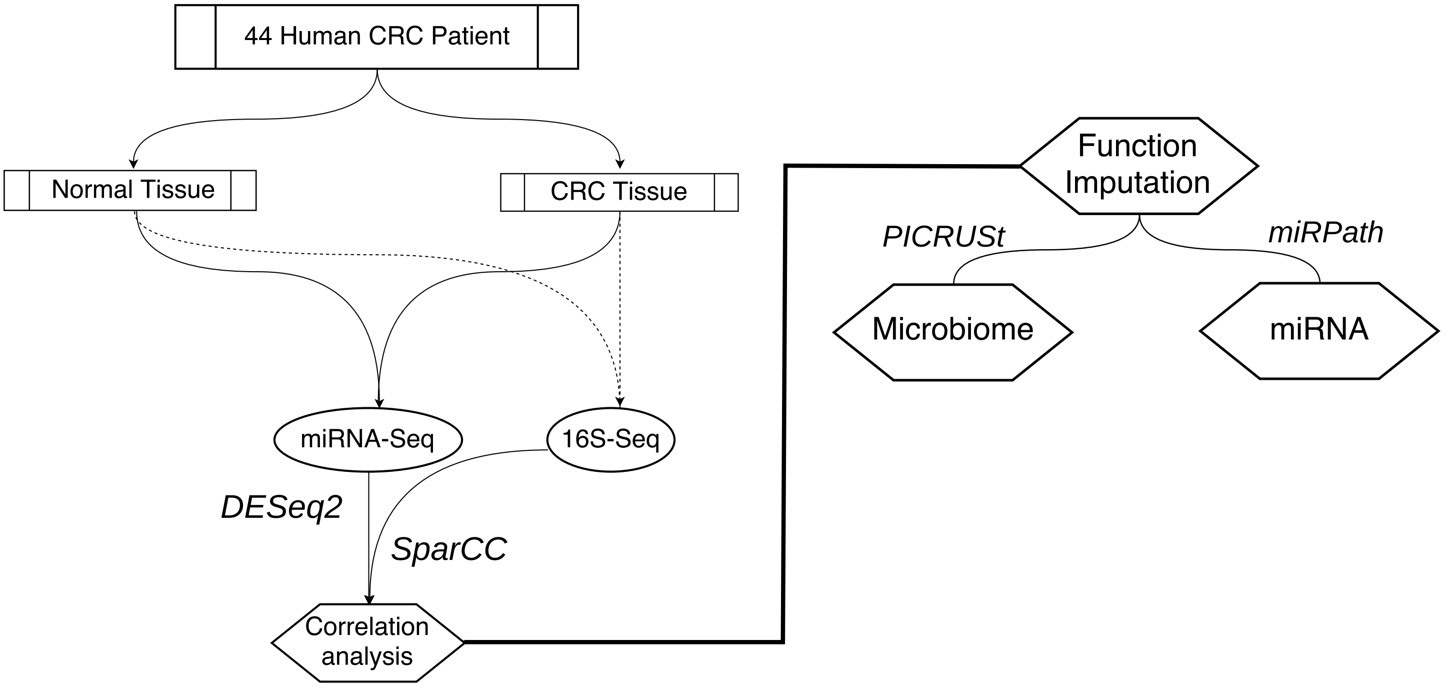

Supplementary Fig. 1. Overview of analysis. Paired tumor and normal tissues were collected from 44 CRC patients by the University of Minnesota BioNet as a part of a previous study. The 16s rRNA gene sequencing was performed prior, as a part of the previous study. The small RNAs were sequenced on an Illumina HiSeq 2000 platform. A detailed description of the analysis was described in methods section.
