# Supplementary material for: Interaction between Host MicroRNAs and the Gut Microbiota in Colorectal Cancer
Source: mSystems. 2018 May 15;3(3):e00205-17. doi: 10.1128/mSystems.00205-17 (PMC5954203; doi:10.1128/mSystems.00205-17)
Supplement: FIG S2 [file sys003182230sf2.pdf]

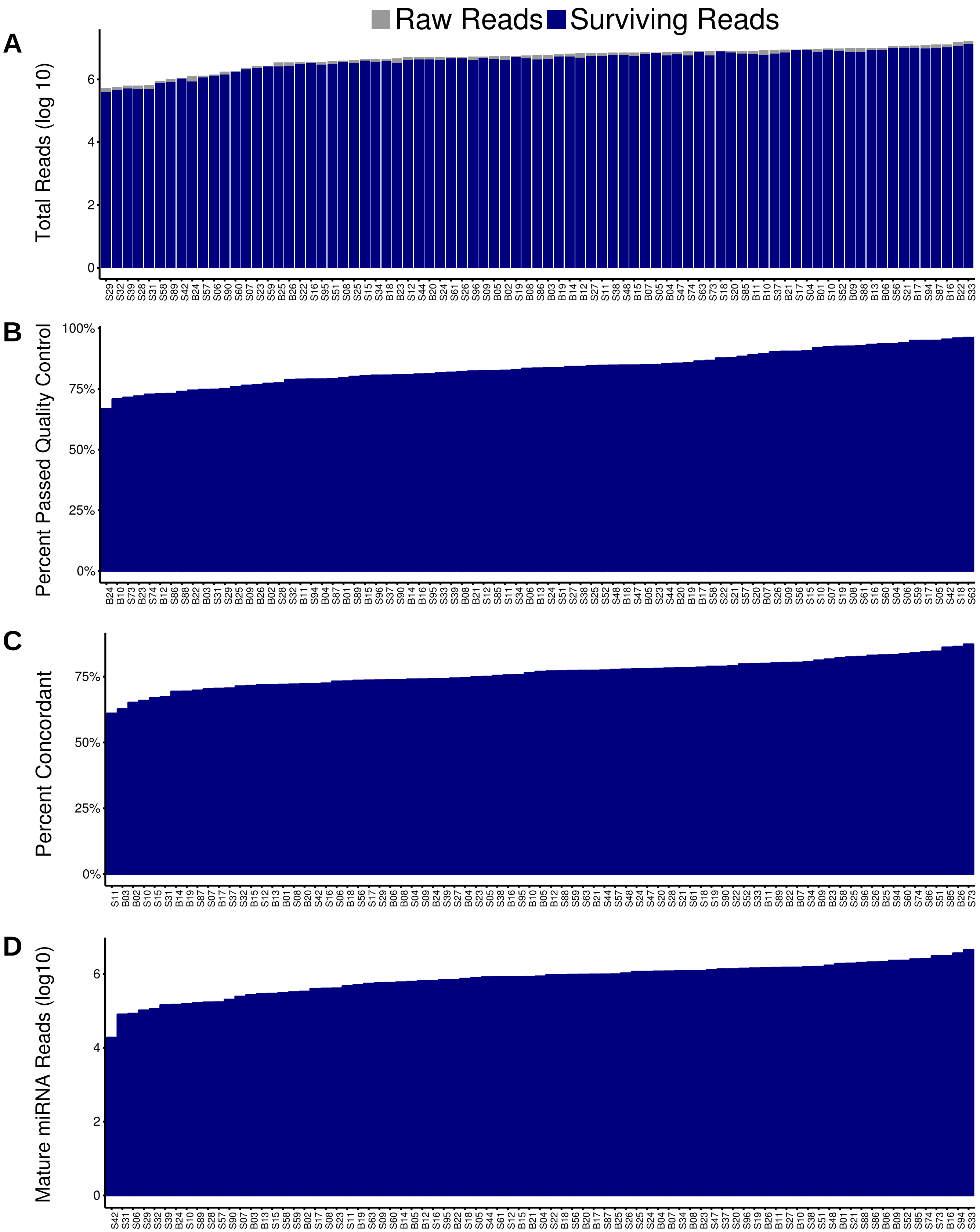

Supplementary Fig. 2. Bar plot of quality control of raw reads. **a.** Total number of raw reads on log10 scale per sample (grey) and percent of reads surviving quality control (blue). **b.** Percent of reads surviving quality control per sample. Bar plot of **c.** percent of concordant paired-end reads per sample and **d.** total number of mapped mature miRNAs per sample on log10 scale.
