# Supplementary material for: Interaction between Host MicroRNAs and the Gut Microbiota in Colorectal Cancer
Source: mSystems. 2018 May 15;3(3):e00205-17. doi: 10.1128/mSystems.00205-17 (PMC5954203; doi:10.1128/mSystems.00205-17)
Supplement: FIG S3 [file sys003182230sf3.pdf]

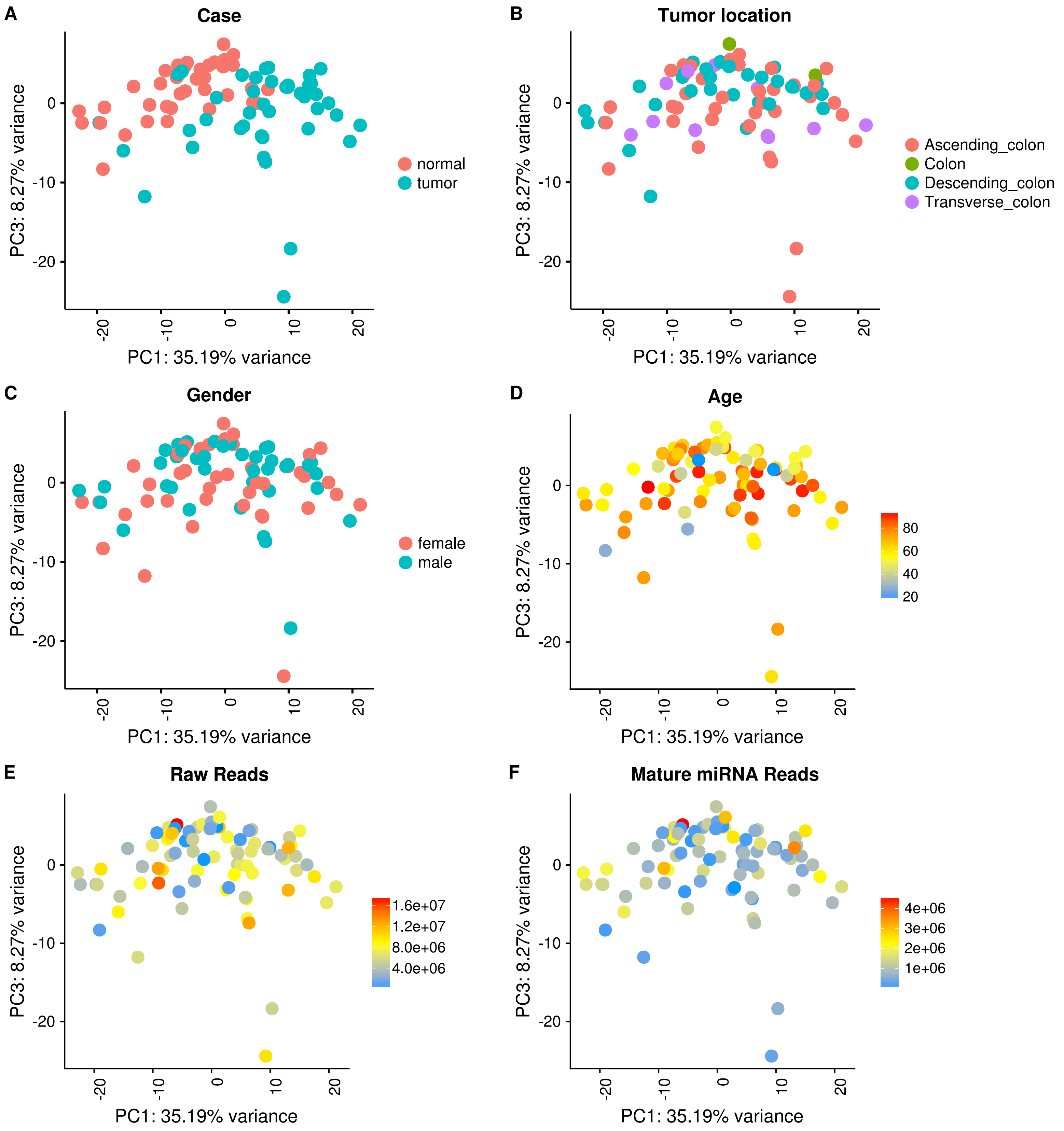

Supplementary Fig. 3. Principle Component Analysis (PCA) plot showing PC1 on x-axis and PC3 on y-axis. Each dot was colored by **a.** Case, **b.** Tumor location and **c.** Gender, **d.** Age, **e.** Raw reads and **f.** Mature miRNA Reads.
