# Supplementary material for: Interaction between Host MicroRNAs and the Gut Microbiota in Colorectal Cancer
Source: mSystems. 2018 May 15;3(3):e00205-17. doi: 10.1128/mSystems.00205-17 (PMC5954203; doi:10.1128/mSystems.00205-17)
Supplement: FIG S4 [file sys003182230sf4.pdf]

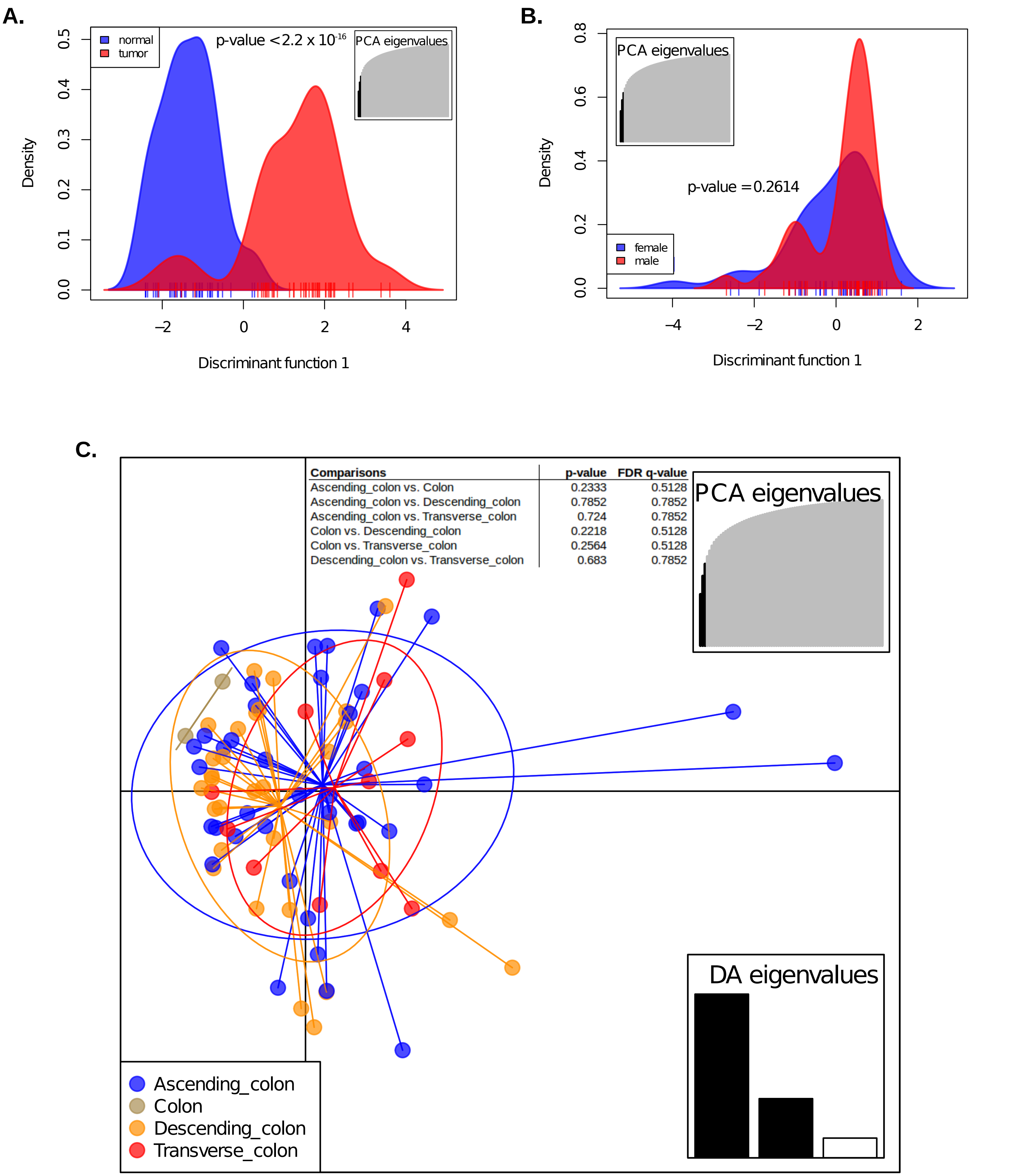

Supplementary Fig. 4. Discriminant analysis of principal components (DAPC) using the first 3 principle components. **a.** Case status show a clear separation between the tumor and normal groups. **b.** Genders and **c.** Tumor location do not affect the does not appear to significantly impact the miRNA expression. Statistical significances are tested using two-sample Kolmogorov-Smirnov test, multiple comparisons are adjusted using false discovery rate (FDR) method.
