# Supplementary figures and images for: Interaction between Host MicroRNAs and the Gut Microbiota in Colorectal Cancer
Source: mSystems. 2018 May 15;3(3):e00205-17. doi: 10.1128/mSystems.00205-17 (PMC5954203; doi:10.1128/mSystems.00205-17)

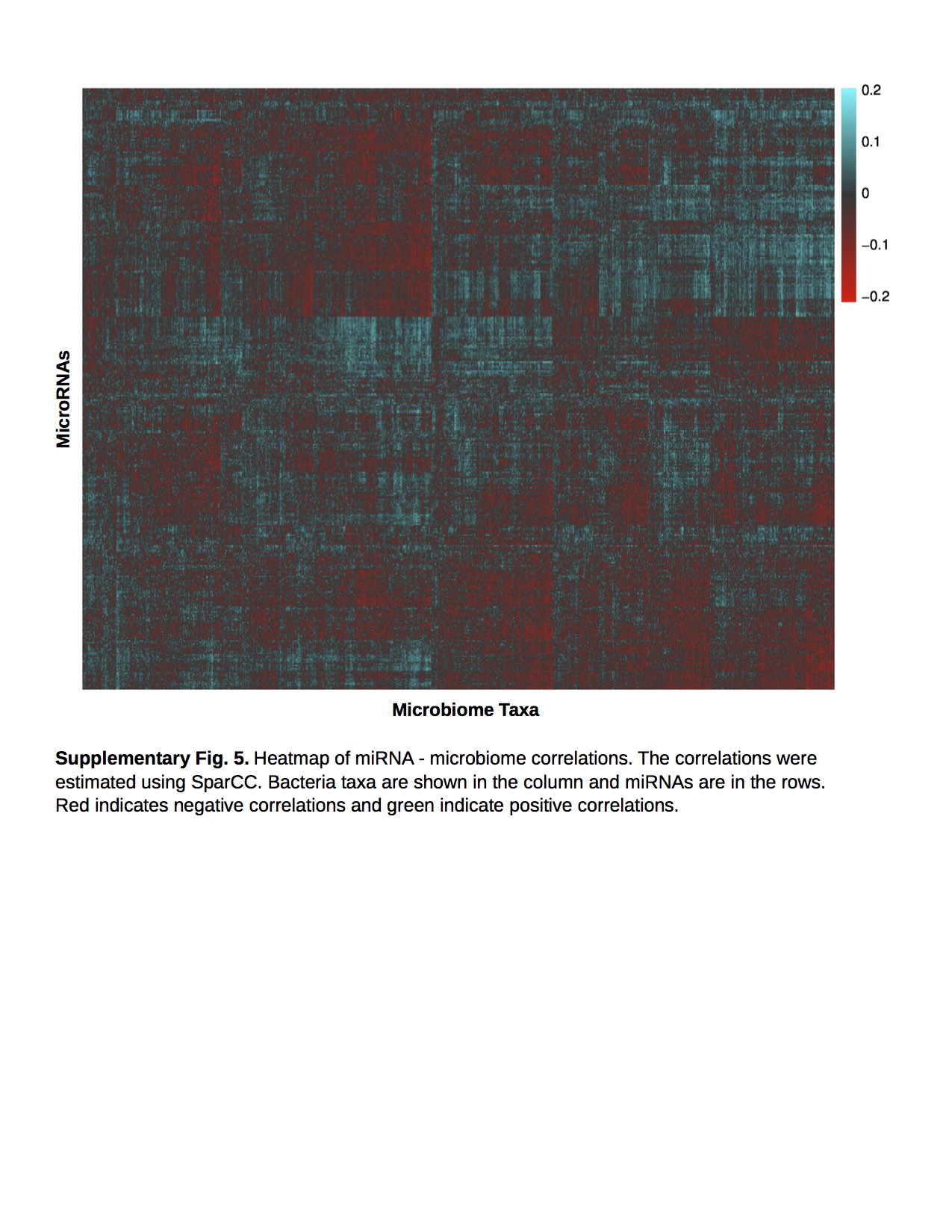

Supplement: FIG S5 [file sys003182230sf5.jpg]
