# Supplementary material for: Interaction between Host MicroRNAs and the Gut Microbiota in Colorectal Cancer
Source: mSystems. 2018 May 15;3(3):e00205-17. doi: 10.1128/mSystems.00205-17 (PMC5954203; doi:10.1128/mSystems.00205-17)
Supplement: FIG S6 [file sys003182230sf6.pdf]

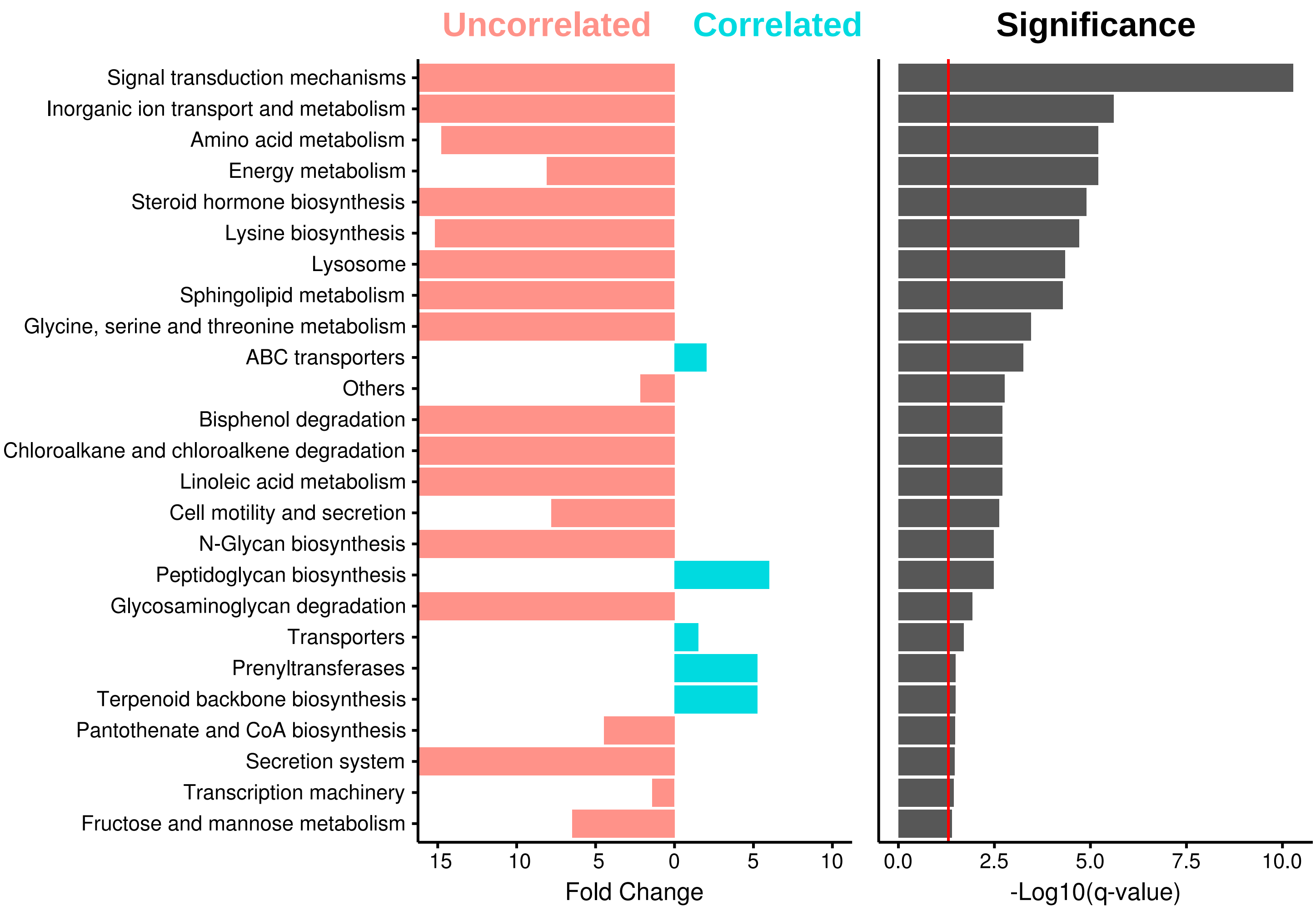

Supplementary Fig. 6. Metabolic pathway (KEGG) enrichment of microbiome correlated and uncorrelated with DE miRNAs. The bar graph (left panel) shows the fold enrichment for each group. Red indicates correlated and blue indicates uncorrelated KEGG enrichment. FDR corrected p-value from Wilcoxon Rank Sum test (on a negative log10 scale) are shown on the right panel. The solid red line indicates q-value of 0.05.
